# Alu-directed transcriptional regulation of some novel miRNAs

**DOI:** 10.1186/1471-2164-10-563

**Published:** 2009-11-30

**Authors:** Tong J Gu, Xiang Yi, Xi W Zhao, Yi Zhao, James Q Yin

**Affiliations:** 1 National Laboratory of Biomacromolecules, Center for Computing and Systems Biology, Institute of Biophysics, Chinese Academy of Sciences, 15 Datun Road, Beijing 100101, PR China; 2 Bioinformatics Research Group, Institute of Computing Technology, Chinese Academy of Sciences, No 6 Kexueyuan South Road, Zhongguancun Haidian District, Beijing 100190, PR China; 3 Graduate School of the Chinese Academy of Science, 19A Yuquan Road, Beijing 100080, PR China

## Abstract

**Background:**

Despite many studies on the biogenesis, molecular structure and biological functions of microRNAs, little is known about the transcriptional regulatory mechanisms controlling the spatiotemporal expression pattern of human miRNA gene loci. Several lines of experimental results have indicated that both polymerase II (Pol-II) and polymerase III (Pol-III) may be involved in transcribing miRNAs. Here, we assessed the genomic evidence for Alu-directed transcriptional regulation of some novel miRNA genes in humans. Our data demonstrate that the expression of these Alu-related miRNAs may be modulated by Pol-III.

**Results:**

We present a comprehensive exploration of the Alu-directed transcriptional regulation of some new miRNAs. Using a new computational approach, a variety of Alu-related sequences from multiple sources were pooled and filtered to obtain a subset containing Alu elements and characterized miRNA genes for which there is clear evidence of full-length transcription (embedded in EST). We systematically demonstrated that 73 miRNAs including five known ones may be transcribed by Pol-III through Alu or MIR. Among the new miRNAs, 33 were determined by high-throughput Solexa sequencing. Real-time TaqMan PCR and Northern blotting verified that three newly identified miRNAs could be induced to co-express with their upstream Alu transcripts by heat shock or cycloheximide.

**Conclusion:**

Through genomic analysis, Solexa sequencing and experimental validation, we have identified candidate sequences for Alu-related miRNAs, and have found that the transcription of these miRNAs could be governed by Pol-III. Thus, this study may elucidate the mechanisms by which the expression of a class of small RNAs may be regulated by their upstream repeat elements.

## Background

MicroRNAs (miRNAs) are a class of small non-coding RNAs (ncRNAs) about 22 nt in length. They control fundamental cellular activities such as differentiation, proliferation, apoptosis and others in different species by regulating gene expression [1-3]. Although miRNAs were discovered more than a decade ago, their transcription remains insufficiently understood. They are believed to be transcribed by polymerase II (Pol-II) [4-6]. However, new research on ncRNA transcription indicates that polymerase III (Pol-III) may participate in this process [7-9]. Pol-III is usually recognized as transcribing housekeeping ncRNAs and short interspersed nuclear elements (SINEs) such as tRNAs, 5s-rRNAs and Alu [7,10,11]. In 2004, a study revealed that the exogenous Pol-III promoter can initiate miRNA transcription [12]. Since then, several lines of evidence have shown that Pol-III can transcribe miRNAs downstream of tRNAs, Alu and other SINEs [7,13,14], but whether this is a common mechanism is still not clear.

In the haploid human genome of three billion base-pairs, the sequences of protein-encoding genes constitute about 3%, whereas repeats and transposons constitute up to 45%. Alu elements are among the most abundant transposons, constituting 11% of the human genome [15]. Alu is about 300 nt in full length, including left and right arms with Poly A sequences between them and at the end [16]. More importantly, it affects genome recombination, RNA transcription, alternative splicing, translation, DNA replication and methylation, and other processes [16,17]. Alu insertion may cause many diseases [18,19]. Therefore, Alu has gradually attracted more and more attention and has been extensively studied in relation to transcription. It is generally believed to be transcribed by Pol-III through internal promoters, the A box and B box [20,21]. Because Alu does not code for a terminator, Pol-III usually reads through its sequence until it reaches a downstream terminator [22,23]. Thus, Pol-III may transcribe sequences downstream of Alu elements.

Therefore, if miRNAs follow Alu elements closely or reside within Alu, they are very liable to be transcribed through Alu by Pol-III. Moreover, it has been demonstrated that Alu can serve as a promoter for miRNA transcription [14]. It has also been found that Pol-III transcribes small RNAs through tRNAs or tRNA-like sequences in Trypanosomatid protozoa, nematodes and plants [24-26], while in the human virus murine gammaherpesvirus 68 (MHV68), Pol-III transcribes downstream miRNAs through tRNA [13]. tRNAs differ from Alu in sequence but are similar in transcription. They both have the A box and B box that are recognized and bound by Pol-III [7,27]. It is reasonable to presume that Pol-III can transcribe other ncRNAs downstream of Alu elements or other repeats. Taking Alu as an example, we propose the hypothesis that the transcription of a class of new miRNA genes can be linked to their upstream Alu transcription, and on this basis we have conducted a group of comprehensive studies.

## Results

### Process of prediction

**Figure 1:**
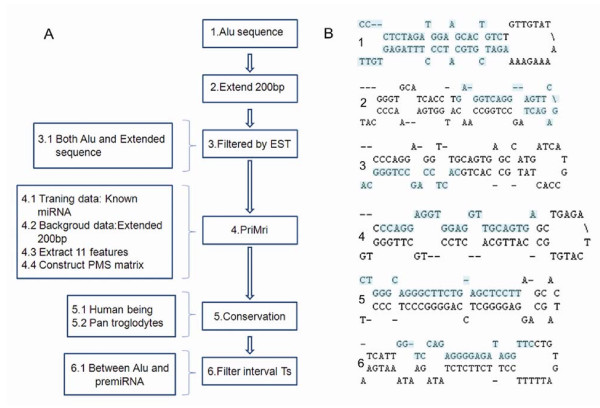
**Schematic representation of the workflow and secondary structures of new miRNA candidates**. A. Schematic representation of the workflow used to predict and verify new miRNAs downstream of Alu elements. B. Secondary structures of new miRNA candidates. The highlighted letters in the foldback structures represent the mature miRNA sequence. 1, 2, 3, 4, 5 and 6 denote miRNAs with ID:AluSg/x-1110454, ID:AluSq/x-374792, ID:AluJo-576611, ID:AluJo-135090, ID:MIRb-367839, and ID:MIR-128218, respectively.

We investigated the number of miRNAs that may reside within the region downstream of Alu elements in the human genome using a newly-developed approach [28]. First, we downloaded all the repeat sequences annotated by the Repeatmasker from UCSC http://genome.ucsc.edu/, and from this we extracted about 1,180,972 Alu sequences and their extensions (200 bp). It is well known that the full length of Alu is about 300 bp, while sequences that can be transcribed by Pol-III are about 500 bp [8]. So, a 200 bp sequence downstream of Alu may be transcribed by Pol-III. Secondly, all the Alu elements and extended sequences not collected in EST were filtered. The resulting 86,966 sequences were found to be transcribed. Thirdly, we predicted 259 miRNAs at the threshold value of 7 using the PriMir software described briefly in the methods section [28]. Two hundred and nineteen sequences remained after the elimination of one nucleotide overlapping with the exon. Alignment of sequences from phylogenetically related species showed that 60 miRNAs and their upstream Alu sequences were highly conserved in primates (Additional file 1). Since Alu is conserved only in primates, those sequences that are conserved between *Pan troglodytes *and humans were selected. When sequences with more than four consecutive Ts (generally known as the Pol-III terminator) between the Alu elements and miRNA genes were eliminated, 60 sequences remained, as shown in the Additional file 1. The entire process is shown in Figure 1A. The secondary structures of some pre-miRNA candidates are illustrated in Figure 1B. Mature miRNA can be generated from either or both arms of a pre-miRNA.

### Analysis of prediction results

**Figure 2:**
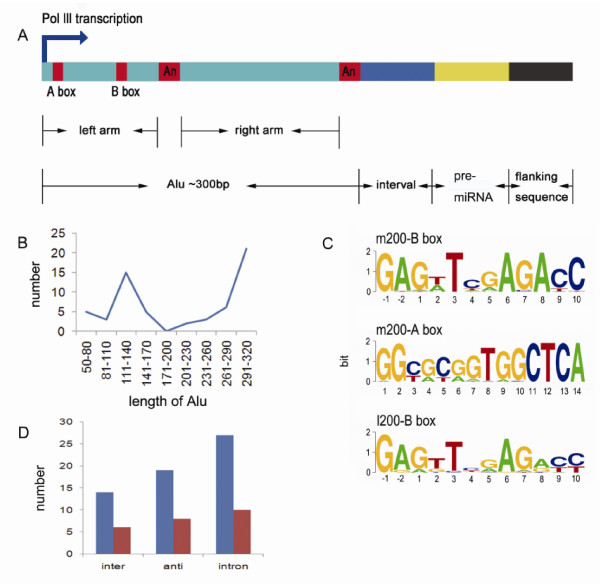
**Bioinformatics analysis of the relationships between Alu and its downstream miRNA genes**. A. Schematic diagram of the structural relationships of Alu elements, miRNA genes and other sequences, suggesting that some miRNAs downstream of Alu elements may be transcribed by Pol-III through an Alu sequence. B. The length distribution of Alu transposons through which miRNAs may be transcribed. The X axis is the Alu transposon length and the Y axis is the number of Alu sequences. C. Alu sequences upstream of miRNA genes are divided into two groups: m200, longer than 200 bp; and l200, shorter than 200 bp. The ATGC content was determined by extracting sequences in line with the A and B boxes from the multiple sequence alignment results. The height of each letter is proportional to the frequency of the nucleotide indicated. Solid letters correspond to the position relative to the starting point of the A or B box. D. The distribution of Alu sequences followed by miRNA genes in the human genome. The abscissa is the location of Alu and miRNA genes: *inter *= sequences between protein-coding genes, *anti *= antisense strands of genes and *intron *= introns. The ordinate indicates the number of sequences.

Analysis of these 60 sequences indicated that the distribution of Alu length had two peaks, one around 120 nt and the other around 290 nt (Figure 2B). These sequences were allocated to two classes: >200 nt, considered the full length of Alu (Additional file 2); and <200 nt, considered to be stable Alu segments inserted in the genome (Additional file 3). The number (n = 28) of >200 nt Alu sequences proved similar to that (n = 32) of the <200 nt Alu sequences. All the AluSc, AluSg and AluY family sequences are longer than 200 nt while those of the FLAM and FRAM families are shorter (Additional file 2 and 3). AluY is the youngest class of the Alu family; AluSg and AluSc are the two youngest classes of the AluS family and are second only to AluY in evolutionary age. FLAM and FRAM are the oldest Alu families [29]. It has been shown that in the young families, the sequences and biological functions of Alu remain comparatively intact, whereas the sequences of the old families such as FAM and AluJo are no longer intact and some have even lost their functions owing to continual mutation during evolutionary history. In contrast, our observation suggested that both young and old members of Alu families may be functional (Additional file 2 and 3). Sequence alignment of the two groups of data showed that most Alu sequences longer than 200 nt basically preserved the A box and B box required for transcription. Only three sequences lacked the B box (Additional file 2). The sequences shorter than 200 bp were all mapped to the left arm of the consensus Alu sequence (Additional file 3). Although sequences shorter than 200 bp no longer had intact A boxes, they basically retained the B box necessary for transcription, as shown in Additional file 3. In contrast, both AluS-126274 (id) and AluJ/FLAM-741298 (id) lacked the B box but retained the A box. It is well known that the A and B boxes, especially the latter, are the internal promoters for Alu transcription [20,21]. This is consistent with our prediction results, showing that both short and long sequences retain the B box. Most of the 60 sequences retained the A box and B box and some sequences around those boxes were also highly conserved. Weblogo analysis (Figure 2C) indicated that the first two nucleotides of the B box (positions -1 and -2) were highly conserved, and G in position -2 was observed in almost all sequences. The sequences following the A box were also found to be similar. For example, positions 11-14 were CTCA in almost all the Alu >200 nt sequences, suggesting that the motif around the A box may be important for Alu transcription.

Of the 60 sequences, 14 were located between protein-coding genes, 19 within the antisense strands of introns and 27 within introns, as shown in Figure 2D. Twenty-seven sequences derived from introns could be mapped to 65 different introns, suggesting that their sources are multiple loci.

### miRNA verification

Of the 60 Alu-related sequences, only one is a known miRNA, miR-517a. This has been shown experimentally to be a functional miRNA that can be transcribed through its upstream Alu [14]. The other miRNAs were newly predicted. To test whether they can be transcribed, we assessed the expression of different types of small RNAs in three different cells. Using high-throughput Solexa technology [30], 4,136,656 total reads and 3,105,897 unique sequences covering 439 known miRNAs were obtained in K562 cells (Additional file 4). Twenty new Alu-related miRNAs were identified. The sequencing results from human embryonic stem cells (hESC) and human embryoid body cells (hEBCs) showed that the total numbers of reads in these two cell types were respectively 6,147,718 and 6,014,187, and the numbers of unique sequences were respectively 5,261,520 and 5,192,421, including 334 known miRNAs ([31]). Another 19 and 10 Alu-related miRNAs respectively were verified. These data indicate that miRNAs that may be transcribed through Alu are abundant in K562 cells and hESCs. Subsequently, the same sequences from the three cell types were integrated, and the numbers of corresponding reads were added. A final total of 24 Alu-related miRNAs were confirmed, including the known miR-517a (Table 1). Mapping between the sequences identified and predicted miRNA genes was shown in Additional file 5. Thus, six newly identified miRNAs were found to reside between protein-coding genes, eight in the antisense strands of introns and 10 within introns (Figure 2D). It is well known that miRNA expression is cell/tissue-specific, development-specific and disease-specific. It is therefore understandable that not all the predicted miRNAs are expressed in the three cell types. If more cell types are used in the future, more predicted miRNAs will be identified.

**Table 1:** The locations of 24 miRNAs downstream of Alu elements in the human genome.

Id-Alu	Chr	Strand	Seq
Intergenic			

AluSx-11437	chr1	+	CTACTCGGGAGGCTGAGGCAGGA
AluSq/x-173806	chr2	+	AGGTCAGGAGTTTGAGACCA
AluSg/x-1110454*	chr19	+	ATCGTGCATCCCTTTAGAGTGTT
AluSx-138854	chr2	-	CCAGGAGGTGGAGGTTGCAGT
AluSc-1117364	chr20	+	GGTGGATCACGAGGTCAGGAG
AluY-346134	chr5	-	GCCACTGCACTCCAGCCTGG

anti-intron			

AluJo-118885	chr2	-	ACGCCTGTAATCCCAGCACTTT
AluSp-1004023	chr17	+	AGGCTGAGGTGGGAGGAT
AluSg-1022676	chr17	+	AGGTCAGGAGTTTGAGACCAGCCTGGCCAA
AluJo-135090	chr2	-	CCAGGAGGTGGAGGTTGCAGTG
AluSx-13507	chr1	-	CGAGACTAGCCTGGCCAACATGGTG
AluJo-576611	chr9	+	CTGCACTCCAGCCTGGGCA
AluSx-3296	chr1	-	GCCACTGCACTCCAGCCTGG
FLAM_C-1110015	chr19	-	TCAGGAGTTTGAGACCAGC

intron			

AluJb-641	chr1	+	AGGCCTGTAATCCCAGCATTTT
AluSx-966613	chr16	-	AGGTGGAGGTTGCAGTGAG
AluSc-109651	chr2	-	ATGCCTGTAATCCCAGCACTTT
AluY-468716	chr7	+	CAGGAGGTGGAGGTTGCAGTGAGC
AluY-1159909	chr22	-	CCAGGAGGTGGAGGTTGCAGTG
AluSg-433754	chr7	+	CTTGAACCCAGGAGGCGGA
AluSg-974039	chr16	+	CTTTGGGAGGCTGAGGTGGG
AluSq/x-374792	chr6	-	GAGGTCAGGAGTTCGAGACT
AluJo-512070	chr8	+	GGAGGCCAAGGTGGGAGGAT
AluY-583205	chr9	-	TACTCGGGAGGCTGAGGCAGGAGAA

The sequences given represent the most abundant and longest miRNA sequences identified by high-throughput solexa sequencing. Asterisk indicates that the pre-miRNA can produce two mature miRNAs. The sequence of another miRNA is: CCCTCTAGATGGAAGCACTGTC.

The same method was also used to explore other repeats. MIR belongs to the same SINE family, and was closely followed by candidate miRNA sequences. Besides the four known miRNAs, a further nine sequences downstream of MIR were verified (Additional file 6). Although the functions of MIR are not yet established, it may have some transcriptional activities. Whether MIR can direct the transcription of its downstream miRNAs awaits further study.

### Verification of expression

**Figure 3:**
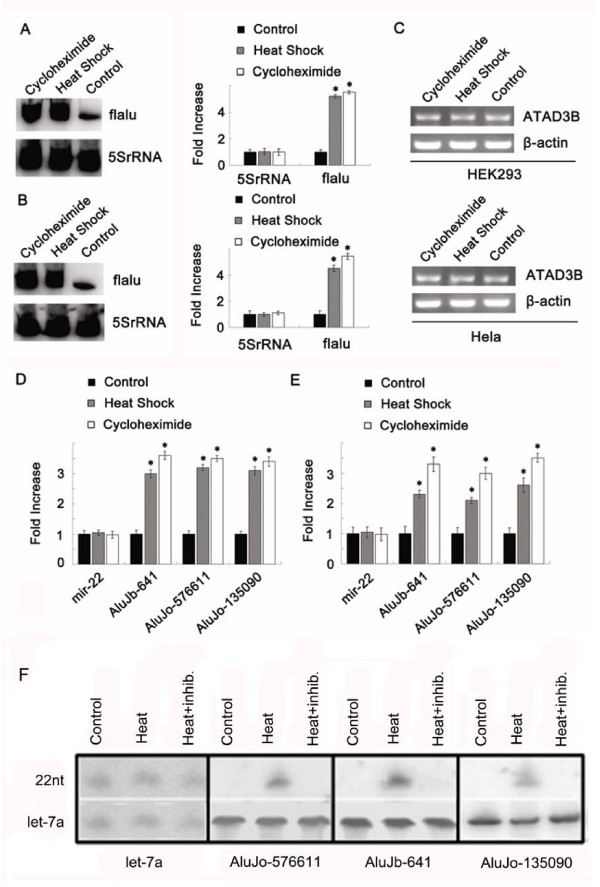
**Cycloheximide and heat shock induce a significant increase in the level of co-expression of Alu and its downstream miRNA**. A. Northern blotting of full length Alu transcripts after induction by heat shock or cycloheximide. Total RNAs extracted from HEK293 cells were separated by urea PAGE and hybridized with flAlu (full length Alu) or 5S rRNA probes. Northern blotting was performed using the Typhoon 9410 Phosphorlmager. The data are averages of at least three independent determinations. The error bars indicate standard deviations. *P < 0.001; cycloheximide or heat shock compared with the control group. B. Northern blotting was used to detect changes in Alu expression in HeLa cells after cycloheximide or heat shock induction. C. RT-PCR of expression of the host gene ATAD3B in HEK293 and HeLa cells after induction by heat shock and cycloheximide. Beta-actin was taken as a loading control. D. After cycloheximide and heat shock induction, the expression levels of Alu-related miRNAs in H293 cells were examined by real-time PCR. 5sRNA was taken as a loading control. The expression levels of miRNA were normalized to that of 5sRNA and the results in different cases were normalized to the expression levels in the control. The results are representative triplicate experiments and are given as mean and S.D. E. After cycloheximide and heat shock induction, real-time PCR was used to detect changes in Alu-related miRNAs in HeLa cells. F. Northern blotting showing inhibition of Pol-III or Pol-II activity by tagetitoxin. Let-7a was taken as both negative and loading control.

From the sequencing results, three representative sequences were randomly selected for serial experiments: AluJb-641, AluJo-576611 and AluJo-135090. AluJo-576611 was found to be located in the antisense strand of *TRIM14*, AluJo-135090 between protein-encoding genes, and AluJb-641 within the seventh intron of the gene encoding mitochondrial membrane protein ATAD3B. To study the relationship between Alu and miRNA expression, we used cycloheximide and heat shock to induce high expression of full-length Alu RNA [32] and then determined whether its downstream miRNAs were also highly expressed. First, Northern blotting was employed to detect changes in the amount of Alu transcripts (Figure 3A and 3B). The results showed that after cycloheximide induction, the levels of full-length Alu transcripts in HeLa and HEK293 cells were elevated 4.7- and 5.1-fold respectively. This observation obtained further support. After heat shock induction, the content of full length Alu transcripts in HeLa and HEK293 cells were elevated 4.2- and 4.8-fold respectively. Real-time TaqMan PCR was then used to detect dynamic changes in miRNAs. After cycloheximide and heat shock induction, small RNAs were reverse-transcribed with a specific Stem-loop RT-primer and quantified by real-time TaqMan PCR. The results showed that in HEK293 cells, AluJb-641, AluJo-576611 and AluJo-135090 were respectively up-regulated 2.19-, 3.43- and 3.66-fold after cycloheximide induction (Figure 3D). Similar results were obtained using HeLa cells (Figure 3E). In contrast, no change in expression of the control miR-22 was detected, strongly suggesting that the expression of these miRNAs is closely related to the expression of their upstream Alu.

To confirm the involvement of Pol-III in miRNA transcription further and exclude the possibility of Pol-II-mediated miRNA generation, HeLa cells were treated with tagetitoxin at a concentration (4 U/ml) that specifically inhibits Pol-III transcription [33]. As shown in Figure 3F, the levels of mature AluJb-641, AluJo-576611 and AluJo-135090 was dramatically enhanced in heat-treated cells, while the level of let-7a, used as a Pol-III negative control, remained unchanged. More importantly, tagetitoxin specifically inhibited Pol-III and resulted in failure to detect mature AluJb-641, AluJo-576611 and AluJo-135090 bands in the 22 nt marker region. However, the transcription of let-7a, which is driven by Pol-II, was not affected by tagetitoxin. This suggests that the transcription of some miRNAs can be regulated by Pol-III and supports our hypothesis that Alu elements may be the promoter specific for nearby downstream ncRNAs.

To clarify whether such intron-derived miRNAs can be co-expressed when their host genes are transcribed, we investigated the expression profiles of AluJb-641 and its host gene. The former was found to reside within the seventh intron of *ATAD3B*, which encodes a mitochondrial membrane protein. This intron was 2,401 bp in full length and AluJb-641 was located at locus 192. RT-PCR showed that expression of the host gene was not enhanced by heat shock or cycloheximide stimulation (Figure 3C), which generally induce high expression of Alu and AluJb-641 (Figure 3A). Thus, AluJb-641 may not be co-expressed with its host gene, but along with the expression of its upstream Alu. Our results also showed that *ATAD3B *was not co-expressed with Alu.

### Identification of a conserved element

**Figure 4:**
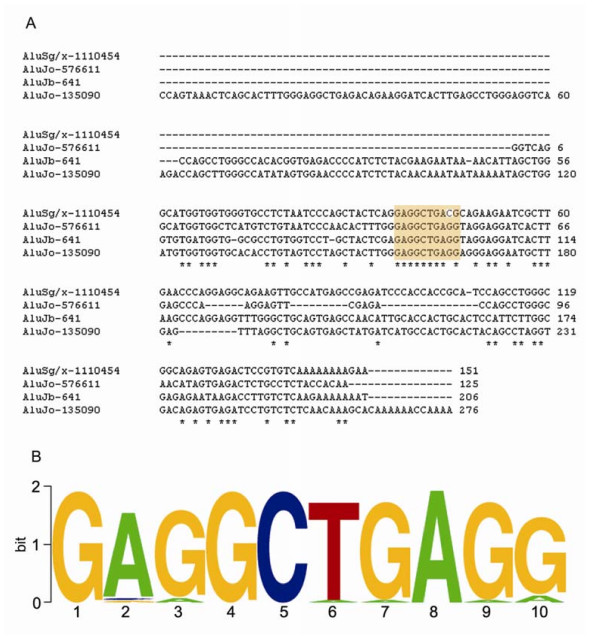
**Conserved motif among different Alu sequences**. A. The multiple comparison of four Alu sequences used for this experiment. B. The ATCG content map illustrates a highly conserved motif, GAGGCTGAGG, shown in the highlight box of A.

Multiple sequence comparisons among three Alus used in this study and AluSg/x-1110454, which resides upstream of miR-517a, revealed that the sequence GAGGCTGAGG was very highly conserved (Figure 4A). Further careful comparison between the sequence GAGGCTGAGG and the consensus sequences of various Alu families revealed that almost all Alu sequences, long or short, contained this sequence. The alignment of Alu sequences (Additional file 2) showed that this motif existed in all the predicted sequences of more than 200 nt (Figure 4B). This suggests that it is highly conserved and may play an important role in Alu transcription or enhance its functions, especially when the A box and/or B box is not intact or deficient.

## Discussion

The effect of Alu on other ncRNAs has rarely been studied. In recent years, as ncRNA research has progressed rapidly, more and more repeats have been found to be important for gene transcription. Dieci et al. comprehensively summarized the evidence that Pol-III may transcribe their downstream sequences through tRNAs, repeats or ncRNAs [7]. On the basis of previous studies, we hereby propose the hypothesis that some short interspersed nuclear elements (SINEs) without terminator sequences may trigger the transcription of their downstream miRNAs. Guided by this hypothesis, we investigated the mechanism of miRNA transcription through Alu (Figure 2A). We not only predicted 60 Alu-related miRNAs that might be transcribed through Alu, including the previously reported miR-517a, but also identified 23 of them by Solexa sequencing. More importantly, using induction by heat shock and cycloheximide, we showed that the expression of Alu RNAs was consistent with the expression of three miRNAs downstream of those elements, providing clear-cut evidence that transcription of some miRNAs may be initiated by Alu transcription.

Recently, studies on miRNA transcription by two different groups have indicated that Pol-II or Pol-III is associated with miR-517a transcription [14,34]. RNA Pol-I is known to be insensitive to α-amanitin; RNA Pol-II is very sensitive to it and RNA Pol-III moderately sensitive. In order to evade the ambiguous effects of α-amanitin, we used tagetitoxin as a specific Pol-III inhibitor [33]. As expected, our results showed that tagetitoxin significantly repressed the expression of Pol-III-mediated miRNAs, but did not affect the transcription of the Pol-II-driven let-7a. Considered together, the inhibitors used for Pol-III may be a key factor in the differences among research results. Although only the example of Alu RNA has been investigated in this article, we cannot exclude the possibility that other SINEs play the same role as Alu, because some known and predicted miRNAs were also found downstream of MIR (Additional file 5). Transcription of these miRNAs seems likely to depend on key sequences such as A/B boxes or other motifs embedded within Alu elements.

Other members of the SINE family, in addition to Alu, all originate from tRNAs and have a classical structure comprising the following three parts: first, a promoter transformed from tRNA; second, a sequence specific for the SINE; third, a sequence needed for reverse transcription, similar to the 3' end of LINE and simple repeats [8,35]. While the other SINE family members have mostly evolved from tRNAs, they are different from Alu in structure but very similar in transcription. It is generally believed that in both tRNAs and SINEs, the A box and B box within the left arm are used to regulate transcription. Of these two boxes, the box B may play a decisive role [7,20,21,27]. Therefore, the transcription of SINE elements is also very likely to trigger the expression of their downstream small RNAs.

However, among the three newly identified miRNAs that have transcriptional activity and are co-expressed with Alu elements, only AluJo-576611 and AluJo-135090 contain the classic B box, and AluJb-641 has no B box; neither has the upstream AluSg/x-1110454, which has been shown to be able to transcribe miR-517a. Even though AluSg/x-1110454 was mapped to the left arm of the uniform sequence of Alu in our multiple sequencing alignments, its B box was severely mutated. In the B box, the critical nucleotides are the first locus G and the third locus T, but the third locus of AluSg/x-1110454 is G. In the multiple sequencing alignments, one motif, GAGGCTGAGG, was found to be highly conserved. It is present not only in these four sequences but also in uniform sequences of all Alu family members. This motif may be of great significance for the transcription of Alu and its downstream miRNAs, or have a similar function to the B box in regulating Alu transcription. However, this possibility still awaits further study.

When miRNAs were discovered, they were originally thought to have nothing to do with repeats. For example, when miRNAs were predicted, Alu-related repeats were first filtered out. Nonetheless, it has been found over the past two years that miRNAs and repeats are closely interrelated. miRNAs are very likely to originate from repeats and may interact with repeats, or with mRNAs with related repeats embedded in their 3'UTR regions [36-38]. Repeats are now known to be able to direct transcription of miRNAs. Considering these findings together, we believe that with further studies, a regulatory network constituted by miRNAs, repeats and target RNAs will be unveiled layer by layer, enhancing our understanding of the nature of life.

## Conclusion

We identified a kind of new miRNAs closely downstream of repeat elements, and found that Pol-III could be involved in transcribing these miRNAs through Alu. Our results elucidated the mechanisms of the Alu-directed transcriptional regulation of some miRNAs and revealed that miRNAs and repeats might be closely interrelated.

## Methods

### Datasets

Most of our data were downloaded from the UCSC table http://genome.ucsc.edu/ in May 2006: repeats from Variation and Repeats, miRNAs and exons from Genes and Gene Prediction Tracks, EST from mRNA and EST tracks. All data were saved in the bed format and uploaded to Galaxy http://main.g2.bx.psu.edu/ for analysis. In view of our knowledge that pre-miRNAs may be located immediately downstream of SINEs, Pol-III-transcribed sequences are usually less than 500 bp in length and most SINE elements are less than 300 bp, we extended 200 bp downstream of SINEs as our initial sequences for predicting candidate miRNA genes.

Human Genome (hg18) and Primate Genome (panTro2) were also downloaded from UCSC using FTP to prepare for conservation analysis.

The consensus sequences of different primate SINE families were downloaded from giri http://www.girinst.org/.

### Filtering by ESTs

To ensure the SINEs were expressed, we compared all SINEs and extended sequences with ESTs. Although most ESTs are transcribed by Pol-II [39], the discovery of non-coding RNA (ncRNA) has led to the inclusion of expressed sequences transcribed by other polymerases in the EST database. Umylny et al. found that 452 Alu ncRNAs longer than 200 bp and probably transcribed by Pol-III are also listed in dbEST [40]. We used a similar method to filter our data except for the differences in SINE lengths. If both SINEs and extended sequences were collected among ESTs, we took them as sequences that are potentially still active.

### Prediction of pre-miRNAs

The software PriMir, developed by us [28], was used to predict miRNAs by the following procedure. First, we created a training data set using known miRNAs and a background data set, using hairpins, extracted from 200 bp extended sequences. Secondly, 11 secondary structure features of known pre-miRNAs were used as screening criteria: (1) the total number of paired bases in the 10-bp up- and down-stream extensions of the pre-miRNA; (2) its total bulge size; (3) the total number of paired bases in it; (4) the length of its loop; (5) the distance between the mature miRNA and the terminal loop; (6) the sequence bias of the first five bases in the mature miRNA; (7) the total number of paired bases in the mature miRNA portion of the pre-miRNA; (8) the minimum free energy (mfe) of the pre-miRNA stem-loop calculated using the RNAfold program; (9) the length of the pre-miRNA; (10) its GC content; and (11) the GC content of the mature miRNA. Using these 11 features, we constructed a PMS matrix. For each entry in the matrix, we calculated the ratio between the frequencies of each feature value in the training and background sets. All entries were calculated according to the definition of (A_ij_). For a given feature i with value j, f_i _(j) and h_i _(j) are the frequencies of this feature in the training and background sets, respectively. X_i _is the feature value set of feature i. If a given j value belongs to X_i_, A_ij _is defined as the log_2 _of f_i _(j)/h_i _(j). Otherwise, A_ij _is assigned the minimum value of *A*_i _x_i_. Then we used PriMir to predict a potential pre-miRNA candidate and calculate a PriMir score from the PMS matrix. The PriMir score value S is defined as the sum of the scores of all features for a given hairpin: Here x_i _is the value of feature i.

Thirdly, we folded the 200 bp extended sequences and extracted pre-miRNA candidates from them on the basis of the 11 features. The reliability of PriMir prediction was evaluated by cross-validation. The training and background sets used to establish the PMS Matrix were divided into five equal parts. Four parts were selected to establish the PMS Matrix, and the remaining part (from both training and background sets) was used to test the performance of PriMir by the ROC curve. The above analysis was repeated five times, each time using a different portion of the data as test data set. A PriMir score of "7" was used as cutoff value. This is a stringent criterion, as ROC curve analysis of the performance of PriMir indicates that the AUC (area under curve) is approximately 0.99, and that the false positive rate is 0 at a PriMir score of 7. Comparisons between PriMir and three other algorithms [41-43] suggested that PriMir was at least equal to and in some respects outperformed those three methods. In addition, we used EST and conservation to filter the initial results, which made our results more convincing. The Accession Numbers for the 24 Alu-related miRNAs deposited at Gene Bank are FJ601661 to FJ601684.

### Conservation analysis

Because Alus are primate-specific repeats and there is only one primate genome, panTro2, in UCSC, we only examined conservation between *Pan troglodytes *and human. In order to find highly conserved miRNAs, we only considered the miRNA candidates with more than 96% similarity to those in *Pan troglodytes*. The lengths of these pre-miRNA candidates in human should be more than 90% of those in *Pan troglodytes*.

We used a similar criterion to find other conserved sequences in SINEs located upstream of pre-miRNAs. The criteria for selection were that the lengths of conserved repeat sequences in *Pan troglodytes *must be more than 90% of those in human and the corresponding similarity must also exceed 90%. If both SINEs and the corresponding pre-miRNAs were conserved and the distance between them was less than 200 nt, we took the pre-miRNAs as our candidates.

### Filtering the candidates

If repeats are expressed in exons of protein-coding genes, they probably do not function as pre-miRNA promoters. Few miRNAs so far known are present in exons, so we filtered out those predicted miRNAs and SINEs that overlapped with exons.

Since more than four Ts between a predicted pre-miRNA and SINE are highly likely to intervene in the transcription of long sequences, we deleted each predicted sequence with more than four Ts between the pre-miRNA and SINE.

### Cell culture

HeLa and HEK293 cells were obtained from ATCC (American Type Culture Collection). The cells were cultured in DMEM (Dulbecco's Modified Eagle Medium) supplemented with 100 U/ml penicillin, 100 U/ml streptomycin and 10% FBS (fetal bovine serum) at 37°C in a humidified atmosphere containing 5% CO_2_. For heat shock experiments, cells were heated in 75 cm^2 ^flasks containing 10 ml medium in a 45°C water bath. After 30 min heat shock, they were incubated at 37°C for 1 h before total RNA was extracted. For cycloheximide treatment, the cells were grown in the presence of 100 μg/ml cycloheximide. The incubation time was 3 h for HeLa cells and 6 h for HEK293 cells prior to total RNA extraction.

### Inhibitors of transcription

Tagetitoxin, a specific inhibitor of RNA Pol-III, was obtained from Epicentre Technologies (Madison, WI). HeLa cells were treated with 120 μmol/l tagetitoxin for one day and then by heat shock as described above. Total RNA was extracted from tagetitoxin-treated and untreated cells. Mature miRNA was isolated and amplified by real-time PCR as described below. The effects of tagetitoxin on in vivo transcription were assessed by Northern blotting. Inhibition of RNA Pol-III was evaluated by the levels of transcription of three Alu-associated miRNAs, with Let-7a as a negative control.

### Small RNA cloning and sequencing

We employed Illumina sequencing methods as described previously. Briefly, total RNA was extracted from human cells with Trizol, and the small RNAs were size-fractionated, purified by polyacrylamide gel electrophoresis (PAGE) to enrich for molecules in the range 18-30 nt, and ligated sequentially to 5'- and 3'-end RNA oligonucleotide adapters. The samples were used as templates for cDNA synthesis. The cDNA was amplified over 15-18 PCR cycles to produce sequencing libraries using Illumina's small RNA primer set. The purified PCR products were subjected to Solexa's proprietary sequencing-by-synthesis method on the Illumina 1G Genome Analyzer. All sequencing services were provided by the Beijing Genomics Institute, Shenzhen http://www.genomics.org.cn and the Genome Sciences Centre, Vancouver. Raw data from IlluminaGA were processed using the initial stages of the Solexa software pipeline (Illumina). Low quality reads were trimmed using perl script. Adaptor sequences were accurately clipped using a dynamic programming algorithm. After redundancy was removed, sequences ≥ 18 nt were mapped to the human genome (UCSC hg18) using SOAP. Small RNA sequences were mapped to human shRNA hairpins by BLAST. The count sum of all isoforms of a shRNA gene was used to measure its expression level.

### Reverse transcriptase reactions

Total cellular RNA was isolated with TRIzol reagent (Invitrogen) according to the manufacturer's instructions. Reverse transcriptase reactions contained 1 μg purified total RNA, 50 nM stem-loop RT primer (Applied Biosystems), 1× RT Buffer, 0.25 mM each dNTP, 5 U/μl M-MLV reverse transcriptase (Promega) and 0.25 U/μl RNase inhibitor (Promega). The mixtures were incubated in a thermocycler for 30 min at 16°C, 30 min at 42°C and 10 min at 85°C, then held at 4°C. All reverse transcriptase reactions, including controls, were run in triplicate.

### Real-time PCR

Real-time PCR was performed using a standard TaqMan MicroRNA Assay kit protocol on a Roter-Gene 6000 (Corbett) Sequence Detection System. The 20 μl PCR reactions included 1 μl RT product, 1× Real-time PCR Master Mix including TaqMan probe, 0.15 μM miRNA specific primer set. The mixtures were incubated at 95°C for 3 min, followed by 40 cycles at 95°C for 15 s and 60°C for 40 s. All reactions were run in triplicate. The threshold cycle (CT) was defined as the fractional cycle number at which the fluorescence passed the fixed threshold. TaqMan CT values were converted to absolute copy numbers using a standard curve from synthetic miRNA Standard.

### Northern blotting

Total RNA was separated by 8% urea PAGE and transferred to a Hybond-N+ membrane (Amersham). The membrane was UV crosslinked, dried thoroughly at 80°C and incubated with hybridization solution (6×SSC, 5×Denhardt's, 0.5% SDS, 100 μg/ml Yeast RNA) and probes labeled with [γ-^32^P] ATP using T4 polynucleotide kinase. After 20 h hybridization at 58°C, the membrane was washed twice at room temperature and once at the hybridization temperature with 20×SSC and 0.1% SDS. Let-7a was taken as a loading and negative control. The bands were autoradiographed and quantified with a Typhoon 9410 Phosphorlmager (GE Healthcare) (Table 2).

**Table 2:** Oligonucleotides used for Northern blotting

Name	Sequence 5'->3'	Position
Alu	GCGATCTCGGCTCACTGCAAG	238-218
5SrRNA	AAAGCCTACAGCACCCGGTATT	120-99
ATAD3B-sen	GGATTCCGTGCCTTTGTGA	807-825
ATAD3B-anti	TTGGTGTTCCTGGTTGCTA	1099-1117
AluJb-641	AAAATGCTGGGATTACAGGCCT	
AluJo-576611	TGCCCAGGCTGGAGTGCAGT	
AluJo-135090	CACTGCAACCTCCACCTCCTGG	
Let-7a	AACTATACAACCTACTACCTCA	

## Authors' contributions

TG carried out the prediction of miRNAs, participated in the sequence alignment, performed the statistical analysis, and drafted the manuscript. XY carried out the molecular genetic studies and Northern blotting. XZ participated in the sequence alignment and coordination. YZ participated in the development of related software. JY designed the study and wrote the manuscript. All authors read and approved the final manuscript.

## Supplementary Material

Additional file 1 Sixty predicted miRNAs downstream of Alu elements. Click here for file

Additional file 2 Alignment of Alu elements longer than 200 nt. Click here for file

Additional file 3 Alignment of Alu elements shorter than 200 nt. Click here for file

Additional file 4 Known miRNAs detected in K562 cells. Click here for file

Additional file 5 The relationships between predicted miRNA and its mature sequences identified by Solexa sequencing. Click here for file

Additional file 6 The known miRNAs and predicted miRNAs identified by Solexa sequencing site downstream of MIRs. Click here for file
